# Supplementary figures and images for: Monosynaptic Tracing Success Depends Critically on Helper Virus Concentrations
Source: Front Synaptic Neurosci. 2020 Feb 14;12:6. doi: 10.3389/fnsyn.2020.00006 (PMC7033752; doi:10.3389/fnsyn.2020.00006)

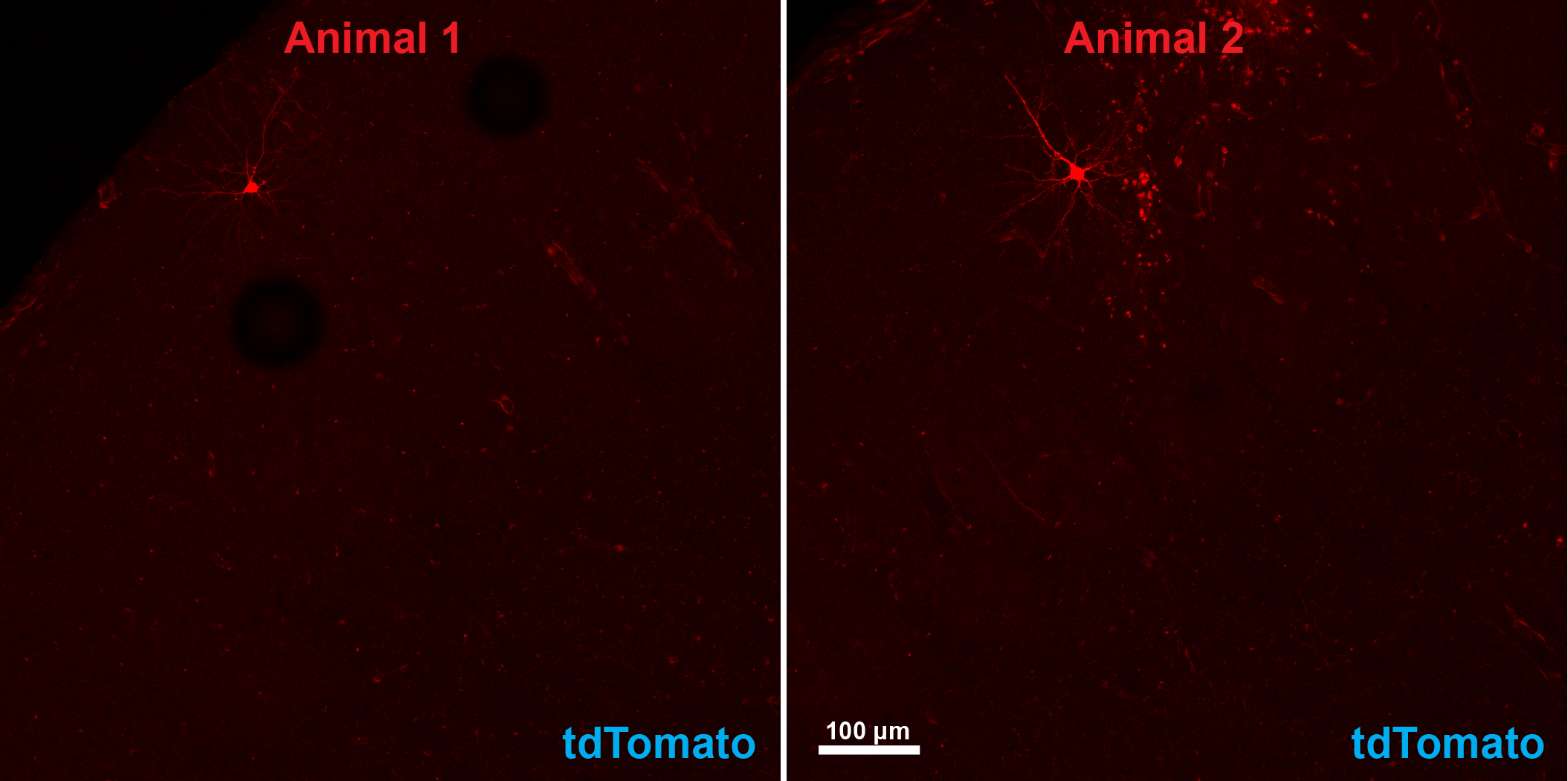

Supplement: FIGURE S1 — Results with rabies virus injection only without helper virus. S1 cortex of two animals were injected with 100 nl RVΔG-4FLPo(EnvA) virus only, without a previous AAV injection. Very few tdTomato-labeled cells were found. Scale bar in a: 100 μm, applies to all panels. [file Image_1.tif]
